# Factors Associated With Medication Safety Competence Among Clinical Nurses in China: A Latent Profile Analysis

**DOI:** 10.1097/jnr.0000000000000749

**Published:** 2026-05-19

**Authors:** Ping ZHANG, Genzhen YU, Rong XU, Shuai CAO, Lin MO, Yang LIU, Yujie WU

**Affiliations:** 1 Department of Nursing, Tongji Hospital, Tongji Medical College, Huazhong University of Science and Technology, Wuhan, China; 2 Department of Nursing, Xiangyang Central Hospital, Xiangyang, China; 3 Outpatient Department, Children’s Hospital of Chongqing Medical University, Chongqing, China; 4 Department of Hematological and Oncology, Children’s Hospital of Chongqing Medical University, Chongqing, China

**Keywords:** medication safety, clinical nurses, medication safety competence, latent profile analysis

## Abstract

**Background:**

Nurses can promote medication safety. However, the main factors affecting their ability to do so are uncertain.

**Purpose:**

In this study, a person-centered approach was employed to analyze the latent profiles of medication safety competence in clinical nurses in China and to identify the significant influencing factors.

**Methods:**

A multicenter cross-sectional online survey was conducted. From January 17 to February 18, 2024, 1,228 nurses (mean age=33.52, *SD*=7.55; 97.20% female) were recruited from several hospitals in the Chinese provinces of Hubei, Chongqing, and Guangzhou and enrolled as participants. The Critical Thinking Diagnostic Scale and Medication Safety Competence Scale were used in collecting study data. Mplus 8.3 software was adopted for the latent profile analysis to clarify the medication safety competence subgroups. SPSS 24.0 software was employed to perform the analysis of variance (ANOVA), Pearson’s Chi-square analysis, and logistic regression analysis to examine the factors associated with medication safety competence.

**Results:**

Four profiles were derived from the latent profile analysis: Profile 1 (low medication safety competence; 9%), Profile 2 (medium low medication safety competence; 8%), Profile 3 (medium high medication safety competence; 40%), and Profile 4 (high medication safety competence; 44%). The total critical thinking score was identified as a positive predictor of profile affiliation. The results of the regression analysis found that, compared with Profile 4, being a junior nurse (*OR*=2.341, 95% CI [1.005, 5.450]), having no self-perceived nursing achievement (*OR*=6.205, 95% CI [2.084, 18.473]), and occasionally having self-perceived nursing achievement (*OR*=7.495, 95% CI [3.419, 16.430]) increased the likelihood of being in Profile 1; that having no history of medication error (*OR*=0.408, 95% CI [.177, .938]) decreased the likelihood of being in Profile 1; that having 11–15 years of work experience (*OR*=2.239, 95% CI [1.166, 4.299]) and occasionally having self-perceived nursing achievement (*OR*=3.102, 95% CI [1.074, 8.953]) increased the likelihood of being in Profile 3; and that being a supervisor nurse (*OR*=0.404, 95% CI [.242, .728]) decreased the likelihood of being in Profile 3.

**Conclusions:**

Based on the results, the medication safety competence of nurses in China may be divided into four subgroups. Having a higher critical thinking score, a professional title (supervisor nurse or above), self-perceived nursing achievement (often/always), and no history of medication error all increase the likelihood of being in Profile 4. The findings provide a reference for nursing managers when formulating continuing education to promote medication safety competence. The factors associated with the various profiles identified in this study may facilitate the development of better-tailored intervention strategies.

## Introduction

Unsafe medication practices and medication errors are the main causes of harm and preventable medication-related injuries in health care systems worldwide (World Health Organization [WHO], 2024). The findings of an updated review indicate that 5% of patients in the health care system suffer preventable drug-related injuries, with patients in low- and middle-income countries almost twice as likely to experience this type of harm than those in high-income countries (7% vs. 4%; WHO, 2024), a prevalence higher than found in the original systematic review (approximately one in 30; Hodkinson et al., 2020). Medical error is the third most common cause of death in the United States after heart disease and cancer (Makary & Daniel, 2016). The early detection of medication errors and prevention of medication-related harm has been highlighted by the WHO as an international priority.

Globally, drug-related injuries impose a significant financial burden on the health care system. The estimated annual cost related to medication errors worldwide is $42 billion (WHO, 2024). An estimated 7,000 to 9,000 people in the United States die each year from medication errors, and ~1.3 million experience medication-related harm (Wittich et al., 2014). Medication-related harms considered “absolutely preventable” have been estimated to cost the National Health Service in the United Kingdom £98 million per year, use 181,626 bed days, and cause or contribute to 1,708 deaths. Medication-related harms include both those occurring in primary care that lead to hospital admissions (£83.7 million, 627 deaths) and those occurring in secondary care that lead to prolonged hospital stays (£14.8 million; causing or contributing to 1,081 deaths; Elliott et al., 2021). The WHO declared the goal of Medicines without Harm to be reducing avoidable serious drug-related harm by 50% globally over the next 5 years.

Medication errors arise from a complex interplay of systemic and individual factors. Systemic failures such as inadequate staffing, suboptimal physical and organizational environments, ineffective communication channels, and underdeveloped incident reporting cultures create conditions conducive to errors (McBride-Henry & Foureur, 2006). Concurrently, individual-level contributors include knowledge gaps in pharmacotherapy, nonadherence to clinical protocols, and prolonged shift durations (Aljuaid et al., 2021; Webb et al., 2025). Health care practitioners consistently identify insufficient staffing levels and excessive workload as primary drivers of medication errors (Alshaikh et al., 2013). Psychosocial stressors, including work–life imbalance, interprofessional conflicts, time constraints, and mandatory night/weekend duties, further amplify error risks through heightened job strain (Salam et al., 2019). Notably, both clinicians and patients perceive off-hours care delivery as inherently risk-prone due to reduced staffing and cognitive fatigue (Miller et al., 2010).

Nurses play an important role in ensuring medication safety, which has been defined as “activities to protect against unintentional harm during the administration of medication and to avoid, prevent, or correct adverse drug events that may result from the administration of medication” (Park & Seomun, 2021). Nurses are at the forefront of advocating for patient rights and serve as the last line of defense in complex medical environments (Thelen, 2022). They must make safe decisions based on interdisciplinary collaboration and take appropriate action to provide safe care (Wilson et al., 2016). Medication safety competence directly affects the practice of unsafe medication behaviors by nurses (N. Zhang et al., 2022), with high medication safety competence mitigating the adverse effects of the medication environment on unsafe behaviors, thus reducing related risks (N. Zhang et al., 2022). Medication safety competence in nurses is vital to minimizing preventable medication-related harm.

A variable-centered approach has been adopted in previous studies to identify the factors affecting medication safety competency in clinical nurses. In the context of clinical nurses, reflective practice has been shown to positively impact the ability to administer drugs safely (Ming et al., 2023), critical analytical disposition has been identified as a positive predictor of medication safety competence (Liu et al., 2023), critical thinking skills have been closely related to medication safety competence (P. Zhang, Gao, et al., 2024), and systems thinking has been shown to relate to patient safety (Hwang & Park, 2017). Future prevention and intervention efforts related to safe medication management targeting nurses in China should be tailored to factors such as gender, hospital level, nature of department, professional position, duties, years of experience, and department (Qin et al., 2023).

Although the variable-centered approach has generated evidence supporting the presence of direct associations between each aspect of medication safety competence and other variables, it has overlooked (a) the existence of different combinations of medication safety competence profiles in the population and (b) differences in these profiles that may correspond to other variables. This perspective aligns with a person-centered approach to conceptualizing medication safety competence, recognizing that various characteristics may exist. Thus, investigating these aspects using a person-centered approach may disclose the impact of drug safety capabilities on other variables.

Latent feature analysis (LPA) is a relatively new approach to addressing a variety of research questions that traditional methods, such as analysis of variance (ANOVA), regression analysis, cluster analysis, and factor analysis, often do not. This approach helps nurse researchers understand the multifaceted relationships, complex patterns, and symptom clusters needed to guide interventions (Ferguson et al., 2019). In this study, a person-centered LPA analysis was used to determine how nurses of different subgroups (i.e., latent classes identified by LPA based on distinct combinations of medication safety competence) utilize distinct combinations of medication safety competence to influence their medication safety performance. The aim of this research was to investigate the current state of medication safety competence among clinical nurses and provide evidence for future interventions to improve related competencies among nurses in China.

## Methods

### Design

A multicenter cross-sectional research approach was adopted, and LPA was employed to analyze the medication safety competence profiles of participating nurses. Differences in sociodemographic and job-related characteristics among the different profiles were also analyzed.

### Participants and Setting

A total of 1,228 nurses from four tertiary hospitals in China were recruited using a convenience sample and enrolled as participants. Nurse managers posted a recruitment notice for this study on the “nursing workgroup” in WeChat, a commonly used social media platform in China. Eligibility criteria included (1) being a registered nurse, (2) >20 years old, (3) participating voluntarily, and (4) being able to complete the survey. Nursing assistants were excluded.

This study was conducted at four tertiary hospitals located in Hubei, Chongqing, and Guangzhou Provinces in China. Tertiary hospitals generally have over 501 beds, are designated at the regional or higher level, provide high-level medical care to multiple regions, and undertake higher education and research.

### Sample Size

The minimum sample size, using a standard deviation (σ=25.13) and margin of error (δ=1.5) of clinical nurses’ medication safety competence, was determined using a literature review (Ming et al., 2023). The type I error probability was estimated as 0.05. The minimum sample size was calculated as 1,078 for this study using the following formula:


$$n={\left(\frac{Z_{1-\alpha /2}\times \sigma }{\delta }\right)}^{2}.$$

### Data Collection

Star Surveys, a popular online survey platform widely used in China, was used to develop the online questionnaire for this study. Online questionnaires are better suited than traditional paper questionnaires because they minimize the occurrence of outliers and data loss. The questionnaire was distributed to all of the nurses who agreed to participate via a WeChat group between January 17 and February 18, 2024. The survey was conducted in a completely anonymous manner, with no personal information collected or disclosed, ensuring participant privacy and confidentiality. The investigator’s assistant uploaded the questionnaire to the WeChat group daily during this time period to ensure all participants received the relevant documents. Also, the participants were instructed not to make duplicate submissions. When reaching the bottom of the dedicated consent page, participants were given the option to continue the survey by clicking on the “I agree to participate” icon. Not clicking on this icon was taken as declining to participate and they could leave the site. Questionnaires were submitted only after all items had been completed and were stored on the online survey platform without any further changes.

### Ethical Considerations

The Clinical Research Ethics Committee of Tongji Hospital approved this study under ethics approval number: TJ-IRB20230904.

### Measures

#### Social and demographic data

The sociodemographic variables collected included age, gender, educational level, years of work experience, professional title, medication error history, self-perceived nursing achievement, and professional interest in nursing.

#### Medication safety competence

The Medication Safety Competence Scale (MSCS) was used to evaluate medication safety competence. This scale was developed and validated by Seomun and Park in South Korea (Park & Seomun, 2021) and translated into Chinese by Yang (Yang et al., 2021). The MSCS consists of 36 items and is scored on a five-point Likert scale (*never* to *always*). Possible scale scores range from 36 to 180, with 36–90, 91–150, and 151–180, respectively, indicative of poor, fair, and good medication safety competence. The six factors addressed in this scale include patient-centered medication management, improvement of safety problems, management of associated factors, management of safety risks, multidisciplinary collaboration, and responsibility as a professional nurse. The Cronbach’s α coefficient for the Chinese version of the MSCS was previously calculated as .940, with domain coefficients ranging from .843 to .94 (Yang et al., 2021), and was .994 in this study.

#### Critical thinking ability

Critical thinking ability was evaluated using the Critical Thinking Diagnostic Scale (CTDS) developed by Berkow (Berkow et al., 2011) and translated and validated by Zhang (P. Zhang, Gao, et al., 2024). The STDS consists of 16 items scored on a six-point Likert scale (from *strongly disagree* to *strongly agree*). Possible scale scores range from 6 to 96, with 0–32, 33–64, and 65–96, respectively, indicative of poor, average, and good critical thinking skills. The five dimensions of the scale include problem recognition, clinical decision-making, prioritization, care plan making, and clinical implementation and improvement. A previous exploratory factor analysis found five factors explained 86.47% of the total variance. The Cronbach’s α coefficient, half-point reliability, and retest reliability of the Chinese version of the CTDS were previously reported as .969, .925, and .835, respectively. In this study, the Cronbach’s α coefficient was .986. Based on this, the CTDS has sufficient validity and reliability (P. Zhang, Gao, et al., 2024).

### Statistical Analysis

The LPA analyses, conducted using Mplus 8.3 software (Muthén & Muthén, Los Angeles, CA, USA), investigated the responses for each CTDS dimension to explore the medication safety competency subgroups. Starting from the two-contour model, the two-, three-, four-, and five-contour models were each tested in succession. Classification accuracy was assessed using the Akaike Information Criterion, Bayesian Information Criterion, adjusted Bayesian Information Criterion (aBIC), and entropy (Nylund et al., 2007), with smaller Akaike Information Criterion (AIC), Bayesian Information Criterion (BIC), and aBIC values associated with better model fit (Tofighi & Enders, 2007) and higher entropy values indicative of better profile classification accuracy (with values ≥0.8 considered good; Celeux & Soromenho, 1996). Also, Lo-Mendell-Rubin (LMR) and bootstrap likelihood ratio test (BLRT) were applied to assess changes in model fit after adding a profile, with *p*<.05 for LMR and BLRT indicating the current contouring model as superior to the previous model (Yin et al., 2020).

Data were processed and analyzed using SPSS 24.0 (IBM Corp., Armonk, NY, USA) to describe sociodemographic and work-related characteristics in terms of frequencies and percentages. Differences in the overall features of the four latent profiles were tested using the chi-square test and ANOVA. Performing LPA before doing stratified multivariate logistic regression may identify potential subgroups based on diverging characteristics, ensuring the regression model takes into account potential heterogeneity and improving the interpretability of the results. This step improves model accuracy by addressing the issue of unobserved population stratification. The multivariate logistic regression analyses conducted in this study included statistically significant independent variables, and a significance level of .05 was considered statistically different for the bilateral tests.

## Results

The 1,228 participants who returned a valid questionnaire ranged in age from 21 to 63 (mean=33.55, *SD*=7.67) years, with the majority between 31 and 40 years old (*n*=535, 43.6%). A plurality had 11–15 years of work experience (*n*=337, 27.4%), and a majority were female (*n*=1,194, 97.2%), had completed university (*n*=1,116, 90.9%), and worked in either pediatric (*n*=384, 31.3%) or internal medicine (*n*=205, 16.7%) wards. Nearly half were junior nurses (*n*=531, 43.2%) and about 20% self-reported as having a medication-related adverse error history (*n*=248).

### Fit Statistics for the Latent Profiles

LPA was conducted on the medication safety competence of the participants, with the results shown in Table 1. For models with 2–5 profiles, we imposed equality constraints on the variances across profiles and set all covariances to zero. In the model comparisons, small AIC and BIC correlate with high entropy, and a BLRT-*p* below .05 indicates better model fit. To clarify the relationship between model selection and fit indices: Although the five-profile model produced numerically lower AIC and BIC values than the four-profile model, this difference was not statistically significant (*p*>.05). Given that the four-profile model also achieved relatively higher entropy and greater parsimony, it was retained as the best-fitting model (Table 1). While the entropy value was highest in the three-profile model, the AIC and BIC were both higher than in the four-profile model. Also, the AIC and BIC of the three-profile model were both lower than in the two-profile model. Therefore, based on the indicators and considerations of model simplicity, the four-profile model was identified in this study as the best model in terms of explanation and additional analysis.

**Table 1:** Fit Statistics for Each Profile Structure (*N*=1,228)

Model	AIC	BIC	aBIC	Entropy	LMR (*p*)	BLRT (*p*)	Proportion
1-profile	39,843.524	39,904.882	39,866.765	—	—	—	—
2-profile	31,942.375	32,039.525	31,979.172	0.990	<.0001	<.0001	0.49/0.51
3-profile	26,628.901	26,761.842	26,679.255	0.994	.0014	<.0001	0.41/0.49/0.10
4-profile	25,433.206	25,601.939	25,497.117	0.993	.0042	<.0001	0.09/0.08/0.39/0.44
5-profile	24,872.732	25,077.258	24,950.201	0.991	.3762	<.0001	0.08/0.37/0.04/0.43/0.08

*Note*. AIC=Akaike Information Criterion; BIC=Bayesian Information Criterion; aBIC=adjusted Bayesian Information Criterion; LMR=Lo-Mendell-Rubin; BLRT=Bootstrap Likelihood Ratio Test; LMR compares a *k*-class model with a (*k*−1)-class model. A significant *p* value (<.05) suggests rejecting the simpler (*k*−1) model in favor of *k* classes, indicating a better fit. BLRT uses parametric bootstrapping to approximate the likelihood ratio distribution. A significant BLRT (*p*<.05) implies the *k*-class model fits significantly better than (*k*−1). BLRT is computationally intensive but robust for complex models. Both tests aid in determining optimal class numbers, though BLRT is generally more reliable.

### Distribution of Medication Safety Competence in the Four-Profile Model

As shown in Table 2, the mean scores of medication safety competence dimensions significantly differed among the four profiles (all *p*<.05). Profile 1 (*n*=112, 9%) had the lowest scores of medication safety competence. Profile 2 (*n*=94, 8%) had low-to-moderate scores of medication safety competence. Profile 3 (*n*=481, 39%) had moderate-to-high scores of medication safety competence. Profile 4 (*n*=541, 44%) had the highest scores of medication safety competence. Based on these results, Profiles 1, 2, 3, and 4 were labeled as “low medication safety competence,” “medium low medication safety competence,” “medium high medication safety competence,” and “high medication safety competence.”

**Table 2:** Distribution of Medication Safety Competence in the Four-Profile Model (*N*=1,228), Standard Mean (*SD*)

Dimension	Profile 1 (*n*=112)	Profile 2 (*n*=94)	Profile 3 (*n*=481)	Profile 4 (*n*=541)	*F*	*p*
	*M* (*SD*)	*M* (*SD*)	*M* (*SD*)	*M* (*SD*)		
Patient-centered medication management (nine items)	3.26 (0.42)	4.01 (0.51)	4.43 (0.26)	4.96 (0.14)	1750.276	<.001
Improvement of safety problems in the medication process (eight items)	3.11 (0.29)	3.96 (0.37)	4.34 (0.17)	4.96 (0.11)	4066.149	<.001
Management of associating factors (six items)	3.10 (0.28)	3.98 (0.32)	4.55 (0.14)	4.98 (0.08)	5600.790	<.001
Management of safety risks (six items)	3.07 (0.26)	3.97 (0.33)	4.63 (0.15)	4.99 (0.06)	6192.598	<.001
Multidisciplinary collaboration (four items)	3.00 (0.33)	3.95 (0.42)	4.46 (0.23)	4.98 (0.11)	3213.625	<.001
Responsibility as a professional nurse (three items)	3.07 (0.38)	3.98 (0.39)	4.65 (0.17)	4.99 (0.10)	3787.718	<.001

*Note*. *M*=dimension mean=total score/number of items. Medication safety competence included six dimensions, namely patient-centered medication management (e.g., communicating individually according to patients’ condition and level in the medication process), improvement of safety problems in the medication process (e.g., improving the complex and vulnerable way of medication safety such as incorrect administration practices), management of associating factors (e.g., understanding the role of human factors, such as fatigue, that related to medication safety), management of safety risks (e.g., coping quickly according to hospital protocol when medication errors or near-misses occur), multidisciplinary collaboration (e.g., collaborating with multidisciplinary professionals to address medication safety issues), and responsibility as a professional nurse (e.g., performing medication care with the alertness as the professional).

### Separate Group Comparisons Between Various Sociodemographic

The sociodemographic variables among the profiles were examined and are shown in Table 3. Significant differences were found across profiles in critical thinking total score, age, working years, professional title, medication error history, self-perceived nursing achievement, and professional interest in nursing (all *p*<.05).

**Table 3:** Comparison of Medication Safety Competence Subgroups by Sociodemographic Variables (*N*=1,228)

Category	Overall (*N*=1,288)	Classification of Latent Profiles (*n*, %)				*F/*χ^2^	*p*
		Profile 1 (*n*=112)	Profile 2 (*n*=94)	Profile 3 (*n*=481)	Profile 4 (*n*=541)		
CT scores (Mean and *SD*)		65.54 (8.80)	83.48 (7.72)	77.73 (5.86)	93.40 (51.50)	950.387	<.001
Gender							
Male	34 (2.8)	1 (0.0)	1 (1.1)	20 (4.2)	12 (2.2)	6.537	.088
Female	1,194 (97.2)	111 (90.1)	93 (98.9)	461 (95.8)	529 (97.8)		
Age (y)							
≤30	483 (39.3)	61 (54.5)	22 (23.4)	228 (47.4)	172 (31.8)	73.463	<.001
31–40	535 (43.6)	42 (37.5)	53 (56.4)	200 (41.6)	240 (44.4)		
41–50	162 (13.2)	8 (7.1)	19 (20.2)	35 (7.3)	100 (18.5)		
>50	48 (3.9)	1 (0.9)	0 (0.0)	18 (3.7)	29 (5.4)		
Years of work							
1–5	177 (14.4)	32 (28.6)	9 (9.6)	87 (18.1)	49 (9.1)	83.620	<.001
6–10	306 (24.9)	29 (25.9)	13 (13.8)	141 (29.3)	123 (22.7)		
11–15	337 (27.4)	34 (30.4)	35 (37.2)	122 (25.4)	146 (27.0)		
16–20	198 (16.1)	8 (7.1)	18 (19.1)	78 (16.2)	94 (17.4)		
>20	210 (17.2)	9 (8.0)	19 (20.2)	53 (11.0)	129 (23.8)		
Educational level							
Associate degree or less	57 (4.6)	5 (4.5)	6 (6.4)	21 (4.4)	25 (4.6)	1.514	.959
Bachelor’s degree	1,116 (90.9)	101 (90.2)	85 (90.4)	440 (91.5)	490 (90.6)		
Master’s degree or above	55 (4.5)	6 (5.4)	3 (3.2)	20 (4.2)	26 (4.8)		
Professional title							
Nurse	186 (15.2)	28 (25.0)	10 (10.6)	89 (18.5)	59 (10.9)	37.887	<.001
Junior nurse	531 (43.2)	51 (45.5)	36 (38.3)	214 (44.5)	230 (42.5)		
Supervisor nurse	472 (38.4)	33 (29.5)	44 (46.8)	169 (35.1)	226 (41.8)		
Chief Nurse	39 (3.2)	0 (0.0)	4 (4.3)	9 (1.9)	25 (4.6)		
Medication error history							
Yes	248 (20.2)	31 (27.7)	24 (25.5)	116 (24.1)	77 (14.2)	22.074	<.001
No	980 (79.8)	81 (72.3)	70 (74.5)	365 (75.9)	464 (85.8)		
Self-perceived nursing achievement							
None	64 (5.2)	15 (13.4)	3 (3.2)	23 (4.8)	23 (4.3)	206.979	<.001
Sometimes feel	662 (53.9)	88 (78.6)	46 (48.4)	317 (65.9)	211 (39.0)		
Often feel	344 (28.0)	9 (8.0)	37 (40.0)	128 (26.6)	170 (31.4)		
Always feel	158 (12.9)	0 (0.0)	8 (8.4)	13 (2.7)	137 (25.3)		
Professional interest in nursing							
Very interested	219 (17.8)	6 (5.4)	17 (18.1)	26 (5.4)	170 (31.4)	216.654	<.001
Interested	769 (62.6)	51 (45.4)	63 (67.0)	346 (71.9)	309 (57.1)		
Not very interested	212 (17.3)	42 (37.6)	12 (12.8)	102 (21.2)	56 (10.4)		
Not interested	28 (2.3)	13 (11.6)	2 (2.1)	7 (1.5)	6 (1.1)	41.840	.074
Unit							
Internal medicine	205 (16.7)	21 (18.8)	15 (16.0)	79 (16.4)	90 (16.6)		
Surgical	122 (9.9)	9 (8.0)	9 (9.6)	40 (8.3)	64 (11.8)		
Obstetrics/Gynecology	58 (4.7)	5 (4.5)	5 (5.3)	18 (3.7)	30 (5.5)		
Pediatric	384 (31.3)	43 (38.4)	31 (33.0)	164 (27.0)	146 (27.0)		
Intensive care unit	35 (2.9)	3 (2.7)	2 (2.1)	22 (4.6)	8 (1.5)		
Operating room	30 (2.4)	0 (0.0)	1 (1.1)	11 (2.3)	18 (3.3)		
Outpatient	76 (6.2)	8 (7.1)	5 (5.3)	28 (5.8)	35 (6.5)		
Oncology	89 (7.2)	5 (4.5)	6 (6.4)	26 (5.4)	52 (9.6)		
Emergency	34 (2.80)	2 (1.8)	4 (4.3)	14 (2.9)	14 (2.6)		
Hemodialysis	41 (3.3)	0 (0.0)	5 (5.3)	15 (3.1)	21 (3.9)		
Others	154 (12.5)	16 (14.3)	11 (11.7)	64 (13.3)	63 (11.6)		

*Note*. CT scores=Critical Thinking total scores. The self-perceived nursing achievements of the participants were self-reported and evaluated using a four-point rating system, divided into no sense of achievement, sometimes feel, often feel, and always feel, respectively represented by scores of 0, 1, 2, and 3.

### Multivariable Logistic Regression of Variables Associated With Latent Profile Membership

A multivariable logistic regression analysis was conducted to examine whether different sociodemographic variables lead to various profiles of medication safety competence among clinical nurses. We used profile membership (Profile 1 to Profile 4) as the outcome variable, with the predictor variables being the total score of critical thinking, age, working years, professional title, medication error history, self-perceived nursing achievement, and professional interest in nursing. Profile 4 was set as the reference group. The final results are shown in Table 4.

**Table 4:** Multivariable Logistic Regression Analysis of Variables Associated With the Latent Profile Membership (*N*=1,228)

Variable	β	*SE*	Wald χ^2^	*p*	*OR* /95% CI
Profile 1					
Total critical thinking scores	-0.570	0.029	375.033	<.001	0.565 [0.0534, 0.599]
Junior nurse	0.851	0.431	3.891	.049	2.341 [1.005, 5.450]
No self-perceived nursing achievement	1.825	0.557	10.754	<.001	6.205 [2.084, 18.473]
Sometimes have self-perceived nursing achievement	2.014	0.400	25.301	<.001	7.495 [3.419, 16.430]
No medication error history	-1.898	0.425	4.455	.035	0.408 [0.177, 0.938]
Profile 2					
Total critical thinking score	-0.216	0.019	127.768	<.001	0.806 [0.776, 0.836]
Profile 3					
Total critical thinking scores	-0.343	0.019	310.843	<.001	0.709 [0.683, 0.737]
Supervisor nurse	-0.906	0.300	9.116	.003	0.404 [0.242, 0.728]
11–15 years of work	0.806	0.333	5.870	.015	2.239 [1.166, 4.299]
Sometimes have self-perceived nursing achievement	1.132	0.541	4.380	.036	3.102 [1.074, 8.953]

The results indicated that the total score of critical thinking, working years, professional title, medication error history, and self-perceived nursing achievement impacted profile membership, while age and professional interest in nursing did not. In the comparison between Profile 1 and Profile 4, higher critical thinking scores, a higher professional title (nurse in charge or above), self-perceived nursing achievement(often/always), and no medication error history were more likely to be associated with Profile 4. In the comparison between Profile 2 and Profile 4, higher critical thinking scores were more likely to be grounded in Profile 4. In the comparison between Profile 3 and Profile 4, higher critical thinking scores, a higher professional title (chief nurse), working more than 15 years, and self-perceived nursing achievement (often/always) were more likely to be associated with Profile 4.

## Discussion

This study provides a new survey of clinical nurses’ medication safety competence through latent profile analysis. During the model fitting process, four latent profiles of nurses’ medication safety competence were identified, namely, “low medication safety competence,” “medium-low medication safety competence,” “medium-high medication safety competence,” and “high medication safety competence.” Most nurses belonged to Profile 4 (44%, high medication safety competence) and Profile 3 (39%, medium-high medication safety competence), indicating that their medication safety competence was at a medium-high level, similar to previous findings (Han et al., 2023; Ming et al., 2023; P. Zhang, Xu, et al., 2024).

Clinical nurses’ medication safety competencies in both Profile 1 and Profile 2 were characterized by low scores on the multidisciplinary collaboration dimension and high scores on the patient-centered medication management dimension. However, Profile 2 scored slightly higher than Profile 1 on both dimensions. The lowest multidisciplinary collaboration score in Profile 1 was related to sociodemographic factors such as younger age, shorter working years, and lower professional title. The majority of nurses in Profile 1 were young, mostly under 30 years of age (54.5%), had been working for <5 years (28.6%), and were junior nurses (45.5%). Nurses with less work experience were more likely to be unprepared and less confident in medication management (Westman et al., 2024), thus reducing the level of multidisciplinary collaboration on medication safety. Moreover, nurses in Profile 1 had a lower interest in the nursing profession (49.2%). Personal interest in a career is an important factor in choosing a major and generating career motivation (Z. Zhang et al., 2023); thus, having a lower interest in the nursing profession may further reduce motivation for multidisciplinary collaboration. Therefore, enhancing medication safety education preparation skills and increasing interest in the nursing profession among young nurses should be encouraged to ensure medication safety in nursing practice.

In Profiles 3 and 4, medication safety competence was characterized by high scores in the dimension of professional responsibility and low scores in the dimension of improving safety problems in the medication process. Over 95% of the participants in Profiles 3 and 4 reported perceiving a sense of accomplishment in their work. Professional identity is linked to a sense of achievement at work (Fitzgerald, 2020), and high perceived accomplishment in practice enhances professional identity in nurses. Professional identity is positively associated with individual team responsibility and individual developmental responsibility (Dong et al., 2013). Thus, a strong professional identity may increase responsibility as a professional nurse. The results of previous studies suggest that improving safety problems is particularly challenging for clinical nurses (Han et al., 2023; Liu et al., 2023). Thus, in China, suboptimal medication safety performance may relate to the late introduction of a nonpunitive culture in relation to adverse care events and to patient involvement in safety management (Guo & Wang, 2012).

In light of the distribution of nurses in each latent profile, medication safety competence differed among the participants due to variations in critical thinking scores, age, working years, professional title, medication error history, self-perceived nursing achievement, and professional interest in nursing. The proportion of nurses aged 30–40 years, working for 11–15 years, holding the title of practitioner, having no medication error history, frequently perceiving nursing achievement, and showing interest in the nursing profession was significantly higher in Profile 4 (high medication safety competence) than in the other subgroups. These findings align with similar studies that identified age, years of working, and professional title as impacting medication safety competence in clinical nurses (Han et al., 2023; Liu et al., 2023). Factors of influence, such as gender, hospital level, unit type, professional title, and years of work, should be targeted in future prevention and intervention efforts targeting safe medication management among nurses in China (Qin et al., 2023). In addition, nurses with a clean medication error history may have higher medication safety abilities than their peers with a history of medication errors. However, this finding is difficult to explain. As reported in previous studies, medication errors may be traced to external factors such as system failures, resource shortages, or communication issues rather than personal shortcomings (Brady et al., 2009). Future research should be conducted to explore the relationship between medication error history and the medication safety abilities of nurses.

Higher critical thinking scores were found to predict greater medication safety competence in this study. Medication safety includes the three attributes of pharmacovigilance, effective skills competence, and interprofessionalism (Thelen, 2022), and, in nursing practice, is a complex process that involves professional knowledge, the practical application of knowledge, technical skills, awareness of patient condition, interpretation of patient data related to medication administration, observational skills, consultation with colleagues, and problem-solving skills (Athanasakis, 2021). Critical thinking, which plays an important role in decision-making and medication competence (Sulosaari et al., 2011), helps clinical nurses analyze complex situations, facilitates safe medication decision-making, and reduces errors in medication decisions (P. Zhang, Xu, et al., 2024). In clinical nurses, critical thinking skills have been shown to be strongly and positively correlated with medication safety competence (P. Zhang, Xu, et al., 2024). Continuing education on medication safety should pay greater attention to improving critical thinking skills in nurses, especially those with low or moderate medication safety competencies.

### Strengths and Limitations

This study was the first to attempt to examine the latent profile of medication safety competence in clinical nurses. The factors found to relate to profile membership in this study may be referenced in the development of more effective medication safety education programs for clinical nurses.

The findings of this study may be limited by several biases. First, online surveys may lack representativeness (e.g., excluding populations without internet access) and data reliability (no interviewer oversight), potentially biasing results. Second, the convenience sampling method used may have introduced selection bias, as the nurses who participated may have been relatively more interested in the research. Third, this study relied on self-reported critical thinking abilities and medication safety competence, which may be subject to recall and social-expectation biases. Fourth, cross-sectional studies cannot track participants over time, limiting the ability to establish causal relationships. Fifth, hospital-level factors were not considered and may affect the results. Thus, controlling for hospital-level factors should be an important consideration in future related research. Finally, the limited number of variables included in this study may not adequately reflect the complexity of the phenomenon being researched. Thus, the findings may not be applicable to all health systems, although they should be relatively more applicable to health systems that share similar characteristics to those addressed in this research.

### Conclusions

Based on potential profile analysis, four profiles of medication safety competence were derived: Profile 1 (low medication safety competence; 9%), Profile 2 (medium low medication safety competence; 8%), Profile 3 (medium high medication safety competence; 40%), and Profile 4 (high medication safety competence; 44%). A high total score for critical thinking, a higher professional title, greater self-perceived nursing achievement, and no history of medication error were identified as positive predictors of high medication safety competence in clinical nurses. Nursing managers should develop tailored continuing education programs based on the factors associated with each profile identified to enhance the medication safety competence of nurses in their teams.

### Implications for Nursing Management

Being a junior nurse and having low levels of critical thinking ability and of self-perceived nursing achievement were found to indicate a greater risk of lower medication safety competence. Efforts are needed to improve medication safety competencies and to incorporate critical thinking skills into interventions tailored to the specific needs of the four identified competency profiles. Young nurses should focus on developing multidisciplinary collaboration skills and nursing professional interests. Improvements in medication safety should be emphasized for senior nurses.

## References

[R1] Aljuaid M. Alajman N. Alsafadi A. Alnajjar F. Alshaikh M. 2021. Medication error during the day and night shift on weekdays and weekends: A single teaching hospital experience in Riyadh, Saudi Arabia. Risk Management and Healthcare Policy, 14, 2571–2578. 10.2147/RMHP.S311638 34188568 PMC8232963

[R2] Alshaikh M. Mayet A. Aljadhey H. 2013. Medication error reporting in a university teaching hospital in Saudi Arabia. Journal of Patient Safety, 9(3), 145–149. 10.1097/PTS.0b013e3182845044 23370218

[R3] Athanasakis E. 2021. Medication safety practices in clinical nursing: Nurses’ characteristics, skills, competencies, clinical processes, and environment. International Journal of Caring Sciences, 14(3), 2019–2028.

[R4] Berkow S. Virkstis K. Stewart J. Aronson S. Donohue M. 2011. Assessing individual frontline nurse critical thinking. Journal of Nursing Administration, 41(4), 168–171. 10.1097/NNA.0b013e3182118528 21430465

[R5] Brady A. M. Malone A. M. Fleming S. 2009. A literature review of the individual and systems factors that contribute to medication errors in nursing practice. Journal of Nursing Management, 17(6), 679–697. 10.1111/j.1365-2834.2009.00995.x 19694912

[R6] Celeux G. Soromenho G. 1996. An entropy criterion for assessing the number of clusters in a mixture model. Journal of Classification, 13(2), 195–212. 10.1097/NNA.0b013e3182118528

[R7] Dong X. Zhao M. Wang W. Li H. Zhang X. Zhou J. 2013. Impact of nurses’ psychological contract on professionl identity. Chinese Journal of Nursing, 48(3), 245–248. (Original work published in Chinese)

[R8] Elliott R. A. Camacho E. Jankovic D. Sculpher M. J. Faria R. 2021. Economic analysis of the prevalence and clinical and economic burden of medication error in England. Bmj Quality & Safety, 30(2), 96–105. 10.1136/bmjqs-2019-010206 32527980

[R9] Ferguson S. L. G. Moore E. W. Hull D. M. 2019. Finding latent groups in observed data: A primer on latent profile analysis in Mplus for applied researchers. International Journal of Behavioral Development, 44(5), 458–468. 10.1177/0165025419881721

[R10] Fitzgerald A. 2020. Professional identity: A concept analysis. Nursing Forum, 55(3), 447–472. 10.1111/nuf.12450 32249453

[R11] Guo X. Wang L. 2012. Improvement of an internet-based voluntary reporting system of nursing errors. Chinese Journal of Nursing, 47(7), 622–624. (Original work published in Chinese)

[R12] Han B. Wei M. Shuao T. Li Q. Wang Y. Xu L. 2023. Current situation and influencing factors of medication safety ability of nurses in secondary and above medical institutions. Chinese Nursing Management, 23(7), 979–983. (Original work published in Chinese)

[R13] Hodkinson A. Tyler N. Ashcroft D. M. Keers R. N. Khan K. Phipps D. Abuzour A. Bower P. Avery A. Campbell S. Panagioti M. 2020. Preventable medication harm across health care settings: A systematic review and meta-analysis. BMC Medicine, 18(1), Article No. 313. 10.1186/s12916-020-01774-9 33153451 PMC7646069

[R14] Hwang J.-I. Park H.-A. 2017. Nurses’ systems thinking competency, medical error reporting, and the occurrence of adverse events: A cross-sectional study. Contemporary Nurse, 53(6), 622–632. 10.1080/10376178.2017.1409081 29166828

[R15] Liu P. Feng Q. Cai P. Zhao R. Yan X. Zhang Y. 2023. Status quo and influencing factors of medication safety competence of clinical nurse. Chinese Nursing Research, 37(17), 3106–3108. (Original work published in Chinese)

[R16] Makary M. A. Daniel M. 2016. Medical error—The third leading cause of death in the US. BMJ, 353, Article i2139. 10.1136/bmj.i2139 27143499

[R17] McBride-Henry K. Foureur M. 2006. Medication administration errors: Understanding the issues. Australian Journal of Advanced Nursing, 23(3), 33–41. 16568877

[R18] Miller A. D. Piro C. C. Rudisill C. N. Bookstaver P. B. Bair J. D. Bennett C. L. 2010. Nighttime and weekend medication error rates in an inpatient pediatric population. Annals of Pharmacotherapy, 44(11), 1739–1746. 10.1345/aph.1P252 20959503

[R19] Ming Y. Xu S. Wu M. Wang J. Fu J. 2023. A study on the impact of reflective practice of clinical nurses on medication safety competence. Chinese Journal of Nursing Education, 20(8), 914–918. (Original work published in Chinese)

[R20] Nylund K. L. Asparouhov T. Muthén B. O. 2007. Deciding on the number of classes in latent class analysis and growth mixture modeling: A Monte Carlo simulation study. Structural Equation Modeling: A Multidisciplinary Journal, 14(4), 535–569. 10.1080/10705510701575396

[R21] Park J. Seomun G. 2021. Development and validation of the medication safety competence scale for nurses. Western Journal of Nursing Research, 43(7), 686–697. 10.1177/0193945920969929 33158408

[R22] Qin N. Shi S. Duan Y. Zhong Z. Xiang G. 2023. Self-reported unsafe medication behaviour among clinical nurses in China: A nationwide survey. Nursing Open, 10(2), 1060–1070. 10.1002/nop2.1373 36177807 PMC9834539

[R23] Salam A. Segal D. M. Abu-Helalah M. A. Gutierrez M. L. Joosub I. Ahmed W. Bibi R. Clarke E. Al Qarni A. A. 2019. The impact of work-related stress on medication errors in Eastern Region Saudi Arabia. International Journal for Quality in Health Care, 31(1), 30–35. 10.1093/intqhc/mzy097 29741703

[R24] Sulosaari V. Suhonen R. Leino-Kilpi H. 2011. An integrative review of the literature on registered nurses’ medication competence. Journal of Clinical Nursing, 20(3-4), 464–478. 10.1111/j.1365-2702.2010.03228.x 20738454

[R25] Thelen M. 2022. Medication competence: A concept analysis. Nurse Education Today, 111, Article 105292. 10.1016/j.nedt.2022.105292 35149327

[R26] Tofighi D., & Enders C. K. (2007). Identifying the correct number of classes in growth mixture models. In G. R. Hancock & K. M. Samuelsen (Eds.), *Advances in latent variable mixture models* (pp. 317–341). Information Age Publishing.

[R27] Webb C. Dabkowski E. Missen K. Missen A. 2025. Contributing factors to medication administration errors among novice registered nurses: An integrative review. Journal of Clinical Nursing, 34(11), 4499–4519. 10.1111/jocn.17721 40033469 PMC12489426

[R28] Westman J. Johnson K. D. Smith C. R. Kelcey B. 2024. Educational preparedness and perceived importance on confidence in new graduate registered nurses’ medication administration. Journal of Professional Nursing, 54, 68–74. 10.1016/j.profnurs.2024.06.008 39266110

[R29] Wilson A. J. Palmer L. Levett-Jones T. Gilligan C. Outram S. 2016. Interprofessional collaborative practice for medication safety: Nursing, pharmacy, and medical graduates’ experiences and perspectives. Journal of Interprofessional Care, 30(5), 649–654. 10.1080/13561820.2016.1191450 27351385

[R30] Wittich C. M. Burkle C. M. Lanier W. L. 2014. Medication errors: An overview for clinicians. Mayo Clinic Proceedings, 89(8), 1116–1125. 10.1016/j.mayocp.2014.05.007 24981217

[R31] World Health Organization. 2024. *Global burden of preventable medication-related harm in health care: A systematic review.* https://iris.who.int/bitstream/handle/10665/376203/9789240088887-eng.pdf?sequence=1

[R32] Yang Z. Chen F. Lu Y. Zhang H. 2021. Psychometric evaluation of medication safety competence scale for clinical nurses. BMC Nursing, 20(1), Article 165. 10.1186/s12912-021-00679-z 34503485 PMC8428106

[R33] Yin K. Peng J. Zhang J. 2020. The application of latent profile analysis in organizational behavior research. Advances in Psychological Science, 28(7), 1056–1070. 10.3724/SP.J.1042.2020.01056

[R34] Zhang N. Kou J. Chang S. Wang Y. 2022. Moderating effect of nurses’medication safety competence on the relationship between medication environment and unsafe medication behaviors. Chinese Journal of Nursing Science, 37(24), 30–34. (Original work published in Chinese)

[R35] Zhang P. Gao C. Qu Q. Wu Y. Wang L. Hu W. 2024. Reliability andvalidity of the Chinese version of the critical thinking diagnostic of frontline nurses. Journal of Nursing Science, 39(1), 64–67. (Original work published in Chinese)

[R36] Zhang P. Xu R. Cao S. Mo L. Liu Y. Gao C. Wu Y. Yu G. 2024. Relationship between critical thinking ability and medication safety competence among clinical nurses: A multicenter cross-sectional study. Journal of Clinical Nursing, 34(6), 2107–2116. 10.1111/jocn.17361 39020523

[R37] Zhang Z. Yang C. Wang Y. Deng G. Chang J. 2023. Investigating the intentions and reasons of senior high school students in registering for nursing education in China. BMC Nursing, 22(1), 311–318. 10.1186/s12912-023-01480-w 37700328 PMC10496206

